# A high glucose diet induces autophagy in a HLH-30/TFEB-dependent manner and impairs the normal lifespan of *C. elegans*

**DOI:** 10.18632/aging.101577

**Published:** 2018-10-05

**Authors:** Berenice Franco-Juárez, Fanny Mejía-Martínez, Elizabeth Moreno-Arriola, Alain Hernández-Vázquez, Saul Gómez-Manzo, Jaime Marcial-Quino, Roberto Arreguín-Espinosa, Antonio Velázquez-Arellano, Daniel Ortega-Cuellar

**Affiliations:** 1 Laboratorio de Nutrición Experimental, Instituto Nacional de Pediatría, Secretaría de Salud, Mexico City 04530, Mexico; 2 Unidad de Genética de la Nutrición, Instituto de Investigaciones Biomédicas UNAM - Instituto Nacional de Pediatría, Mexico City 04530, Mexico; 3 Laboratorio de Bioquímica Genética, Instituto Nacional de Pediatría, Secretaría de Salud, Mexico City 04530, Mexico; 4 Consejo Nacional de Ciencia y Tecnología (CONACYT), Instituto Nacional de Pediatría, Secretaría de Salud, Mexico City 04530, Mexico; 5 Departamento de Química de Biomacromoléculas, Instituto de Química, Universidad Nacional Autónoma de Mexico, Mexico City 04510, Mexico

**Keywords:** high glucose, autophagy, lifespan, HLH-30, calcineurin, *Caenorhabditis elegans*

## Abstract

A high-glucose diet (HGD) is associated with the development of metabolic diseases that decrease life expectancy, including obesity and type-2 diabetes (T2D); however, the mechanism through which a HGD does so is still unclear. Autophagy, an evolutionarily conserved mechanism, has been shown to promote both cell and organismal survival. The goal of this study was to determine whether exposure of *Caenorhabditis elegans* to a HGD affects autophagy and thus contributes to the observed lifespan reduction under a HGD. Unexpectedly, nematodes exposed to a HGD showed increased autophagic flux via an HLH-30/TFEB-dependent mechanism because animals with loss of HLH-30/TFEB, even those with high glucose exposure, had an extended lifespan, suggesting that HLH-30/TFEB might have detrimental effects on longevity through autophagy under this stress condition. Interestingly, pharmacological treatment with okadaic acid, an inhibitor of the PP2A and PP1 protein phosphatases, blocked HLH-30 nuclear translocation, but not TAX-6/calcineurin suppression by RNAi, during glucose exposure. Together, our data support the suggested dual role of HLH-30/TFEB and autophagy, which, depending on the cellular context, may promote either organismal survival or death.

## Introduction

The amount and type of food consumed is a fundamental determinant of human health. Evidence suggests an association between high sugar consumption and the risk of developing metabolic diseases, including obesity and type-2 diabetes (T2D), which eventually lead to decreased life expectancy [1].

Autophagy is an evolutionarily conserved catabolic process that has been linked to both human health and metabolic diseases, such as obesity and T2D [2]. Normally, autophagy is present at a basal level but can be enhanced under metabolic stresses, such as hypoxia, starvation, endoplasmic reticulum stress and lysosomal stress [3], indicating that, depending on the intensity of these stressors, autophagy may be beneficial or detrimental for organismal survival [4]. In fact, it has been shown that genetic hyperactivation of autophagy exacerbates glucose intolerance in mice fed a high fat diet; however, this change can be reversed by transient inhibition of autophagy. Therefore, autophagy is considered a double-edged sword that can promote either cell survival or death [5].

Autophagy is transcriptionally regulated specifically by the transcription factor EB (TFEB), which is an evolutionarily conserved factor homolog to the *Caenorhabditis elegans* HLH-30 [6,7]. TFEB/HLH-30 has emerged as a central regulator of autophagy, favoring the transcription of autophagy-related genes (ATGs). However, new evidence has shown that this process can be activated in response to lysosomal and metabolic stress, performing a broad and crucial role in various cellular processes, including lipid oxidation, immune response, apoptosis and lifespan extension. A recent investigation suggested an unknown new role of TFEB as a potent inducer of cell death whose mechanism has not been studied [8–12]. TFEB exerts its transcriptional activity by its nuclear translocation and its phosphorylation state, which may be partially explained by the kinase mTORC1 (the mechanistic target of rapamycin) and the phosphatase calcineurin, which phosphorylate and dephosphorylate TFEB [13], respectively. However, it has been suggested that other kinase and phosphatase (PKD and PP2A) pathways also modulate the nuclear localization of TFEB [14,15].

A high glucose diet is known to decrease the lifespan of a wide range of eukaryotic organisms that include metazoans such as the nematode *C. elegans* [16,17]. However, the molecular mechanism by which high glucose levels decrease lifespan is still unclear. Interestingly, data showed that HeLa cells cultured with the disaccharide sucrose promoted nuclear translocation of TFEB with a subsequent augmentation in the expression of autophagic and lysosomal genes [9,18]; however, whether activation of TFEB in a sucrose-dependent manner has deleterious effects on cellular viability has not been studied, and little is known about the mechanism involved in TFEB nuclear translocation under stress conditions.

In this study, we provide evidence that supports the role of autophagy in an HLH-30-dependent manner to decrease lifespan under a high glucose diet in *C. elegans*. Additionally, we show that nuclear translocation of HLH-30 is dependent on phosphatases unrelated to calcineurin. Together, our data support the suggested dual role of HLH-30/TFEB and autophagy, which, depending on the cellular context, may promote either organismal survival or death.

## RESULTS

### A high glucose diet increases autophagic flux and autophagy-related genes

**Figure 1:**
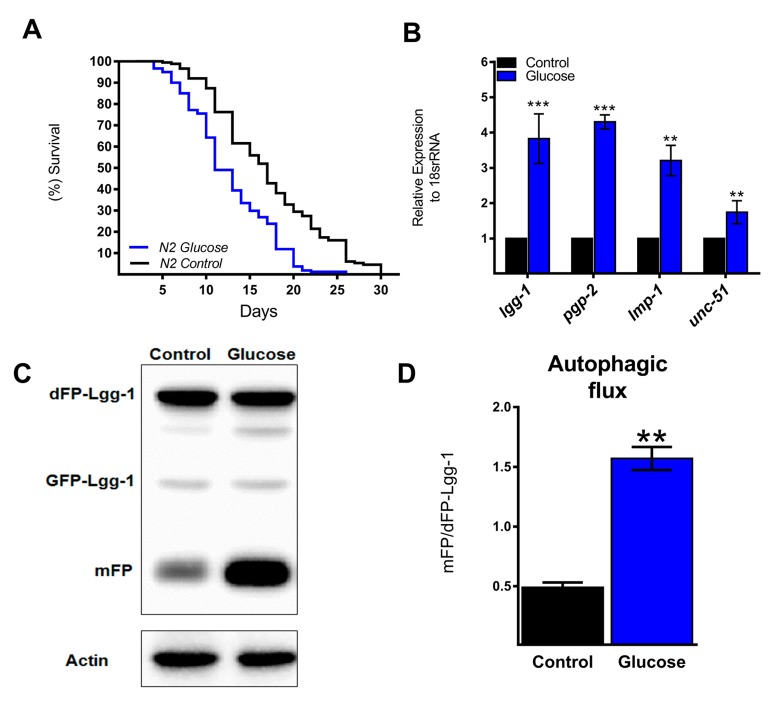
**Autophagic flux and related genes increase with a high glucose diet**. (**A**) Lifespan determined by Kaplan-Meier analysis of N2 wild type animals treated with a high glucose diet showed a decrease in lifespan compared to that of untreated animals. (**B**) Expression of selected autophagic and lysosomal genes measured by quantitative PCR (qPCR) that showed increased mRNA with high glucose. The relative expression of each gene was normalized to that of endogenous 18S rRNA. (**C**) Representative Western blot shows an increase in the band of mFP from the dimeric dFP-LGG-1 when worms were subjected to a high glucose diet. (**D**) The mFP/dFP-LGG-1 ratio indicates an increase in autophagic flux compared to that in normal conditions. *** p < 0.001; ** p < 0.01, Error bars represent ± SEM, t test with Bonferroni’s post hoc test using GraphPad Prism.

Similar to previous studies [16,19,20], wild type nematodes (N2 strain) exposed to a high glucose diet exhibited a significant decrease in their mean and maximal lifespans compared to controls (Figure 1A). Autophagy is considered a cellular process that maintains metabolic homeostasis when organisms are exposed to various stresses. In fact, autophagy in *C. elegans* is primarily considered a pro-survival mechanism [21]. Therefore, we hypothesized that high glucose could negatively affect autophagy. To assess this hypothesis, we first measured the effect of a high glucose diet on the autophagic process by measuring the expression of several ATGs. Unexpectedly, N2 animals fed 100 mM glucose had increased mRNA levels of *lgg-1*, *pgp-2*, *lmp-1*, and *unc-51* (Figure 1B), suggesting that the autophagic process is transcriptionally active under a high glucose diet. To verify whether autophagic flux was also increased, we used a transgenic reporter strain that expressed the LGG-1 protein fused with a dimeric green fluorescent protein (dGFP) [22]; when the autophagic process is active, the dFP-LGG-1 construct is cleaved and consequently releases the protease-resistant mFP and hence indicates an increase in autophagic flux demonstrated by an increase in the mFP/dFP-LGG-1 ratio. As shown by Figure 1C and D, a significant increase in the mFP/dFP ratio was detected in nematodes treated with high glucose, indicating increased autophagic flux.

### High glucose induces activation of HLH-30 and its target genes

**Figure 2:**
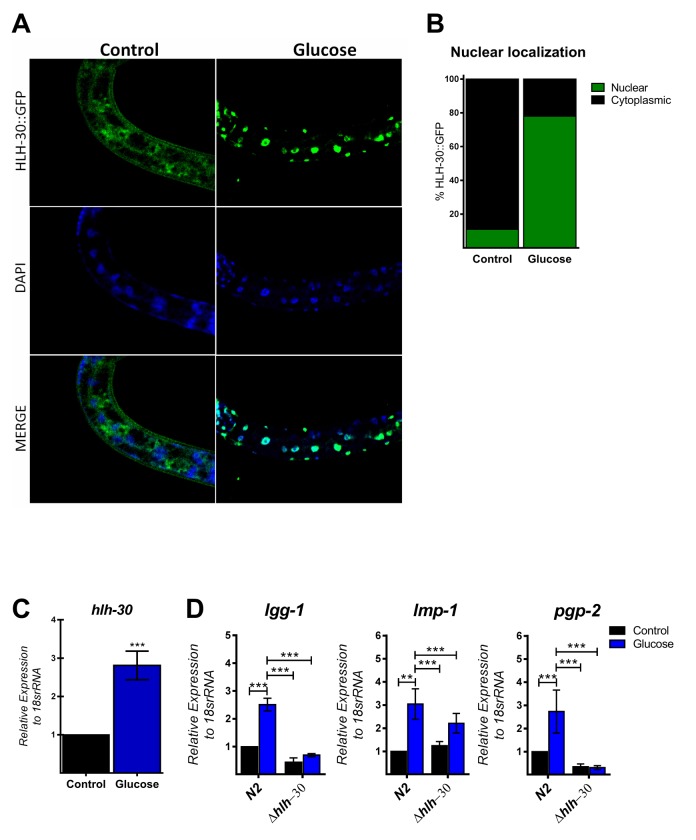
**HLH-30 is activated by a high glucose diet.** (**A** and **B**) Representative confocal images and quantitation, respectively, showing the nuclear localization of HLH-30 with the high glucose diet (green dots). Nuclei were visualized by DAPI staining (blue dots). (**C**) shows the expression level of *hlh-30* mRNA that was increased in wild type nematodes treated with a high glucose diet. (**D**) mRNA expression of putative autophagy-related and lysosomal target genes in the N2 and *hlh-30* (tm1978) mutant strains. Data represent the mean ± SEM of three independent experiments; *** p < 0.001, one-way ANOVA with Bonferroni’s post hoc test using GraphPad Prism.

The autophagic machinery may be transcriptionally activated by HLH-30, a *C. elegans* homolog of mammalian TFEB [23]. Under normal conditions, HLH-30 localizes mainly in the cytosol but rapidly translocates to the nucleus under stress conditions to promote cellular adaptation by upregulating the transcription of autophagic and lysosomal genes [23]. To investigate whether high glucose modified HLH-30 intracellular localization, we used a transgenic HLH-30::GFP reporter nematode strain fed a high glucose diet for 24 h. As shown in Figure 2A and B, a high glucose diet significantly stimulated HLH-30 nuclear localization from the cytosol to the nucleus. Consistent with its proposed autoregulatory feedback loop [11], HLH-30 mRNA increased 3-fold in the N2 strain under the same experimental conditions (Figure 2C). However, a high glucose diet had no effect on the expression of these same genes in the *hlh-30* mutant strain (Figure 1B and 2D). Together, these data revealed that a high glucose diet activates ATGs through the HLH-30-activated transcription factor.

### HLH-30 mutations in glucose-treated worms improve lifespan

**Figure 3:**
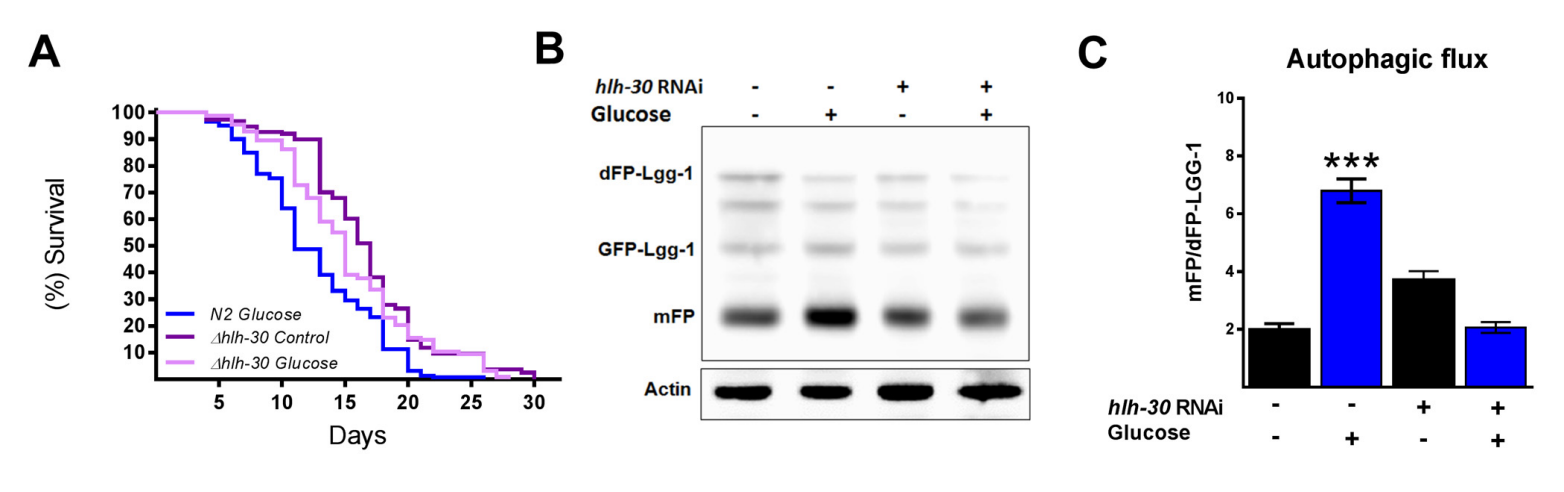
**HLH-30 regulates lifespan and autophagic flux with a high glucose diet**. (**A**) Lifespan analysis of *hlh-30* mutant (tm1978) nematodes showed that they partially rescued the decrease in lifespan induced by a high glucose diet (**B**) Representative Western blot showing a decrease in autophagic flux (less release of mFP from dFP::LGG-1) when *hlh-30* was silenced by RNAi. (**C**) The mFP/dFP-LGG-1 ratio was quantified by densitometry. ***p < 0.001. Error bars represent ± SEM, one-way ANOVA. Images and blots are representative of three independent experiments.

It has been suggested that autophagy and its major regulator TFEB might promote either survival or cell death under certain stress conditions [12]. Therefore, because we observed that HLH-30 and the autophagic process are active under excess glucose, we tested whether the absence of HLH-30 could abrogate the decreased lifespan in high glucose-fed animals. To this end, we grew nematodes of the *hlh-30* mutant strain with a high glucose diet and measured their lifespan (Figure 3A). We found that the absence of *hlh-30* with a high glucose diet allowed the re-establishment of the control lifespan expectancy (Figure 3A). Because both mammalian TFEB and its *C. elegans* homolog HLH-30 are major regulators of autophagy and to better understand the role of HLH-30 in defining the lifespan through the autophagic process, we depleted HLH-30 in nematodes by RNA interference (RNAi), and autophagic flux was analyzed. Accordingly, *hlh-30* RNA silencing (Supplementary Figure 1) significantly reduced autophagic flux since these animals had a diminished mFP/dFP-LGG-1 ratio (Figure 3B and C). These results indicated that HLH-30 is involved in mediating glucose-dependent autophagic flux and might be the major reason for the life limitation in *C. elegans.*

### PP1 and/or PP2A are necessary for glucose-induced HLH-30 activity in *C. elegans*

**Figure 4:**
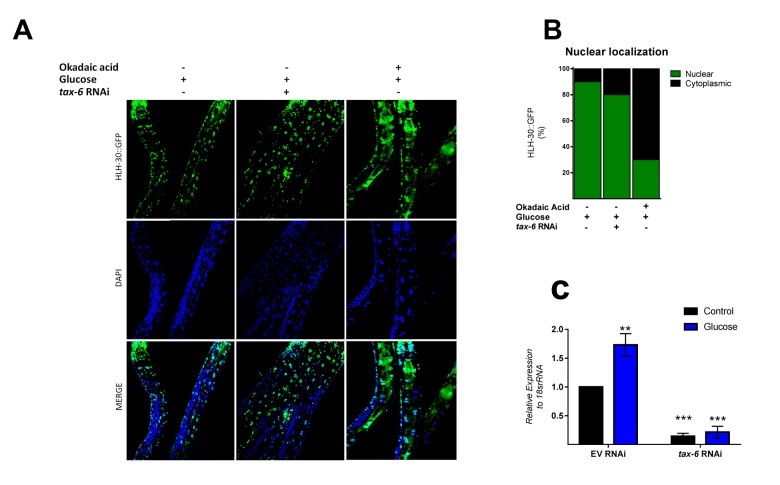
**PPI and/or PP2A might regulate HLH-30 activation.** (**A**) Confocal images of HLH-30::GFP worms treated with high glucose and TAX-6 interference by RNAi did not affect the nuclear localization of HLH-30, whereas pharmacologic addition of okadaic acid (120 nM) prevented it with a high glucose diet. Quantification is given in (**B**). Nuclei were labeled with DAPI (blue dots), (**C**) qRT-PCR analysis of *tax-6* mRNA with or without high glucose after treatment with control (empty vector) or *tax-6* RNAi. p-value (***p< 0.001, **p < 0.01). Error bars represent ± SEM, one-way ANOVA with Bonferroni’s post hoc test using GraphPad Prism.

Because mammalian TFEB activation is achieved by dephosphorylation through calcium-dependent calcineurin protein phosphatase [24], we reasoned that a similar mechanism might be involved in HLH-30 activation. To examine whether TAX-6, an ortholog of mammalian calcineurin A, has a similar effect on HLH-30 localization, we grew the HLH-30::GFP transgenic strain under a high glucose diet and performed a *tax-6* RNAi knockdown. Our results showed that *tax-6* RNAi (Figure 4C) fails to inhibit the nuclear accumulation of HLH-30 in glucose-treated worms (Figure 4A and B), suggesting that HLH-30 activation is a calcineurin-independent mechanism. It has been suggested that other families of phosphatases, specifically PP2Ac, may regulate TFEB activity [15]. To investigate this hypothesis, we simultaneously treated worms with high glucose and okadaic acid (OA), a specific inhibitor of the PP1 and PP2A protein phosphatases [25], at different concentrations (30, 60, 120 and 240 nM) for 24 h and found that the nuclear localization of HLH-30 was modified in a dose-dependent manner (Supplementary Figure 2). Nematodes incubated with 30 and 60 nM of OA displayed nuclear HLH-30 with low amounts in the cytoplasm, whereas at 120 and 240 nM, the localization was preferentially cytosolic (Supplementary Figures 2A and B). Therefore, due to the high cytosolic HLH-30 detected in nematodes treated with 120 nM, we used this OA concentration, that generally is used to evaluate the effect of OA on phosphatase (PP2A) [15], to determine that the localization of HLH-30 is mediated by PP2A protein phosphatase (Figure 4A, right panel) instead of calcineurin/TAX-6 because *tax-6* knockdown did not modify the cellular localization of HLH-30 (Figure 4A, middle panel).

### DISCUSSION

A high glucose diet is known to decrease the lifespan of a wide range of eukaryotic organisms that include metazoans, such as the nematode *C. elegans*, the fruit fly *Drosophila melanogaster*, and various mammals [16,17,19]. However, the molecular mechanism by which high glucose levels decrease lifespan is not well understood. This report provides evidence that supports the role of the HLH-30 transcription factor in lifespan determination under a high glucose diet. We found that (i) a high glucose diet increased HLH-30 nuclear translocation and therefore its activity, which was observed by the induction at the transcriptional level of the ATGs *lgg-1*, *lmp-1*, *pgp-2*, and *unc-51*; (ii) high glucose activates autophagic flux in an HLH-30-dependent manner; and (iii) HLH-30 nuclear translocation is dependent on phosphatases unrelated to calcineurin and concomitantly enhances autophagic genes. The current study provides evidence that the activation of HLH-30-dependent autophagy is implicated in a decrease in lifespan.

TFEB/HLH-30 belongs to the MIT family of bHLH transcription factors and is considered the main regulator of autophagy and lysosomal function [7,26]. Previous studies have shown that TFEB/HLH-30 localizes predominantly in the cytoplasm in basal conditions and translocates into the nucleus upon several cellular stresses, such as lysosomal impairment, bacterial infection, prolonged ER stress, and nutrient scarcity [10–12,27]. In fact, the activation of TFEB/HLH-30 during nutrient deprivation leads to an increase in the lifespan of *C. elegans*; however, recent reports have shown that TFEB could respond in an unexpected manner depending on the type of stimulus [12,23]. Thus, depending on the cellular context, HLH-30 might lead to cell death, suggesting that TFEB/HLH-30 has a new role as a potent inducer of cell death in different stress conditions that have not been studied extensively thus far. Consistent with this idea, a positive relationship between the progressive nuclear translocation of TFEB and the subsequent augmentation in the expression of autophagy and lysosomal genes with high sucrose concentrations has been reported previously in HeLa cells [9,28]. Nevertheless, whether sucrose-dependent TFEB activation has deleterious effects on cellular viability has not been studied, and little is known about the mechanism involved in TFEB nuclear translocation under this stress condition.

Given the different roles of TFEB, we hypothesized that HLH-30 also affects the lifespan under a high glucose diet. Interestingly, we found that with a high glucose diet, HLH-30 increases its nuclear location and transcriptional activity, which correlates with the reduced lifespan of *C. elegans*, since the loss of HLH-30 resulted in partial rescue of lifespan, supporting the notion that HLH-30 activated under high glucose conditions reduces lifespan. This finding is consistent with the fact that activation of mammalian TFEB also promotes cell death, suggesting a conserved role of HLH-30 in *C. elegans* since its activation by glucose decreased the lifespan.

Several lines of evidence suggest that in mammals, calcineurin (PP2B), a serine/threonine protein phosphatase regulated by cellular calcium, activates several transcription factors through dephosphorylation events [24,29,30]. In mammals, calcineurin dephosphorylates and hence activates TFEB, promoting several pathways, such as autophagy, fatty acid oxidation, and immune response, among others. In *C. elegans*, it has been suggested that calcineurin (TAX-6) regulates lifespan through autophagy [31]; however, the mechanism of regulation of TAX-6 has not been identified. Given these findings, we reasoned that TAX-6 might regulate HLH-30 function by modulating its nuclear translocation. Our data showed that nuclear accumulation of HLH-30 with a high glucose diet is TAX-6-independent since RNAi against *tax-6* did not alter HLH-30 nuclear accumulation, suggesting that other phosphatases could regulate HLH-30 nuclear localization. To test this idea, we evaluated the glucose-dependent nuclear localization of HLH-30::GFP with okadaic acid, an inhibitor of the protein phosphatases PP2A and PP1, and we observed a significant dose-dependent decrease in nuclear HLH-30, suggesting that PP2A or PP1 are targets of HLH-30, and confirmed that, as in mammals, HLH-30 is regulated by dephosphorylation events. This result is in concordance with Chen *L* et al., who found that hormone-dependent nuclear localization of TFEB is blocked by okadaic acid and independent of calcineurin [15]. In addition, it is important to highlight that the expression of the protein phosphatase PP2A has been reported to be increased under high glucose conditions [32,33]. Taken together, our data suggest that nuclear glucose-dependent translocation of HLH-30 is regulated by dephosphorylation events dependent on the PP2A or PP1 protein phosphatases in *C. elegans*.

It has been shown that autophagy, a highly conserved catabolic process, plays important roles in many physiological processes, including extension of lifespan and health promotion; however, under certain circumstances, such as prolonged cellular stress, increased autophagy can lead to cell death. Nevertheless, our understanding of the dual role of autophagy in cell survival and cell death remains incomplete. Our data provide evidence that enhanced autophagy is due to a high glucose diet in worms, resulting in a decrease in lifespan. These results are consistent with the observed role of autophagy in switching from advantageous to harmful cellular effects that may contribute to decreased lifespan, supporting the idea of the dual role of autophagy in extending or decreasing the life of multicellular organisms*.* Thus, our data suggest that HLH-30/TFEB-mediated autophagy contributes to limiting the lifespan of *C. elegans* grown with a high glucose diet. These findings indicate that the autophagic process is an active mechanism in the adverse effects of metabolic diseases, such as obesity and diabetes, because of carbohydrate-rich diets.

## MATERIALS AND METHODS

### Strains of *C. elegans*

The *C. elegans* strains used in this study include N2 Bristol (wild type), HLH-30::GFP (MAH235), dFP::LGG-1 (DLM1), and *hlh-30 (*JIN1375). All of these strains were provided by the Caenorhabditis Genetic Center (CGC, University of Minnesota, USA), which is run by the National Institutes of Health-Office of Research Infrastructure Programs.

### Culture, maintenance and experimental conditions

All strains were grown at 20 °C under standard procedures according to Brenner [34] and fed *Escherichia coli* OP50-1 culture for 28–30 hours from the L1 stage. After this time, the worms were moved to NGM control plates (2 g NaCl, 3 g KH_2_PO_4_, 0.5 g K_2_HPO_4_, 0.0085 g/mL cholesterol diluted in 1 mL of absolute ethanol, 30 g Bactoagar, and H_2_O up to 1 L) or to glucose-supplemented plates (100 mM) previously seeded with *E. coli* OP50-1 and supplemented with 49 μM of 5-fluoro-2′-deoxyuridine (FUDR, Sigma-Aldrich). For okadaic acid treatment, nematodes were placed on NGM plates containing 100 mM glucose, and then, okadaic acid (Sigma-Aldrich) was added to these plates to a final concentration of 30, 60, 120 and 240 nM and allowed to dry for approximately 20 minutes. Then, *E. coli* OP50-1 was seeded onto each plate. Worms were exposed for 24 hours to this condition. Three independent experiments were performed with more than 30 worms for each experimental condition.

### Lifespan assays

Lifespan analyses were conducted at 20 °C. Fifty fourth-stage nematode larvae were moved to each experimental condition. They were counted every 1–2 days and were considered dead if they did not respond to a platinum wire loop touch. Nematodes were transferred to new plates every 2 or 3 days or if the plates were contaminated with mold.

### Gene expression analysis

Nematodes of each experimental condition were collected in M9 (6 g Na_2_HPO_4_, 3 g KH_2_PO_4_, 5 g NaCl, 0.25 g MgSO_4_, in 1 L of H_2_O), washed 3 times and flash frozen in liquid nitrogen. Total RNA was extracted by the Proteinase K (Boehringer-Ingelheim) method and purified by TRIzol® as previously described [35,36]. RNA quantification was performed with a NanoDrop® at 260 nm. cDNA was generated using M-MLV reverse transcriptase (Invitrogen™) and random hexamer primers according to the manufacturer’s protocol. Gene expression was determined by RT-qPCR using specific TaqMan® probes for each gene.

### Confocal imaging

Transgenic worms expressing HLH-30::GFP (MAH235) were harvested and washed 3 times with M9 buffer until the bacteria were cleaned. M9 buffer was removed entirely before the addition of DAPI. DAPI was prepared at a concentration of 200 ng/mL in ethanol, and the nematodes were incubated with 10 μL of this solution in darkness until the ethanol evaporated. Next, the worms were rehydrated by adding 1 mL of M9 buffer overnight at 4 °C. For confocal imaging, stained worms were mounted on 2% agarose pads on glass slides under glass cover slips. Fluorescence was examined at 358 nm using a confocal FluoView FV1000 microscope (Olympus) and quantified using ImageJ software (National Institutes of Health, Bethesda, MD, USA).

### RNAi by feeding

*E. coli* HT115 (DE3) strains expressing *tax-6* RNAi (C02F4.2 ORF), *hlh-30* RNAi (W02C12.3 ORF) or the empty vector pL4440 were grown overnight at 37 °C on LB (10 g Bacto-tryptone, 5 g yeast extract, 10 g NaCl) plates supplemented with tetracycline (15 μg/mL) and carbenicillin (2 mg/mL). A single colony of each RNAi clone was taken and inoculated separately in LB with carbenicillin (500 μg/mL) and incubated for 8 hours at 37 °C with constant shaking. A lawn of corresponding bacteria was placed onto NGM plates containing 1 mM IPTG and 25 μg/mL carbenicillin, and these plates were allowed to dry overnight and stored at 4 °C. Synchronized populations of nematodes were grown on these plates until the F2 generation before transferring to experimental conditions. RNAi-validated clones were obtained from Dharmacon©.

### Western blot analysis

Nematodes of DLM1 strain collected in M9 buffer and flash frozen in liquid nitrogen were mechanically homogenized in lysis buffer (HEPES 50 mM, KCl 50 mM, EDTA 1 mM, EGTA 1 mM and Triton X-100 0.1%) supplemented with protease inhibitor cocktail (Complete, Roche). The supernatant was separated by centrifugation (15000 rpm/10 minutes/4 °C), and proteins were quantified by the Bradford method. Then, 50 μg/μL of extracted protein was loaded and separated by SDS-PAGE on a 12% polyacrylamide gel and transferred to a nitrocellulose membrane (Bio-Rad Laboratories). The membrane was placed in blocking buffer (5% fat-free milk in TBS-Tween 20 buffer) overnight and incubated for 1 hour with polyclonal anti-GFP (Life Technologies cat. A21311) or anti-actin antibody (Sigma cat. A2103), which was used as the loading control, at a dilution of 1:500 or 1:2000, respectively, and an appropriate secondary antibody for 1 hour. Monomeric FP (mFP) or dFP proteins were detected by chemiluminescent reactions (ImmobilonTM®). Images were acquired on a FUSION FX System (Vilber Lourmat, France), and data were analyzed with Quantity One 1-D Analysis software (Bio-Rad).

## Supplementary Material

Supplementary Figures
