# Supplementary material for: A high glucose diet induces autophagy in a HLH-30/TFEB-dependent manner and impairs the normal lifespan of C. elegans
Source: Aging (Albany NY). 2018 Oct 5;10(10):2657–67. doi: 10.18632/aging.101577 (PMC6224263; doi:10.18632/aging.101577)
Supplement: Supplementary Figures [file aging-10-101577-s001.pdf]

SUPPLEMENTARY FIGURES

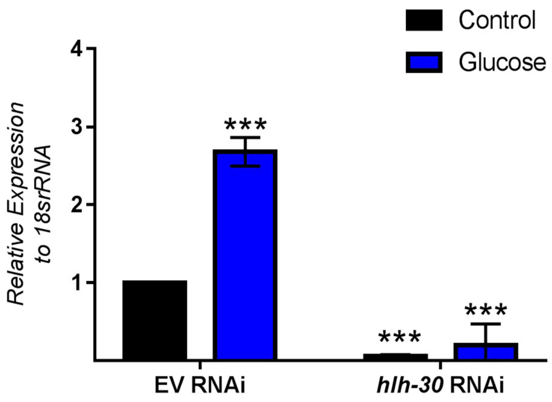

Supplementary Figure 1. mRNA expression of *hlh-30.* qRT-PCR analysis of *hlh-30* expression after treatment with the control (empty vector) or *hlh-30* RNAi. The bars indicate the mean ± SEM relative to nematodes fed with control RNAi from three biological replicates. p-values (*p<0.05 and **p<0.01) were determined by one-way ANOVA with Bonferroni's post hoc test using GraphPad Prism.

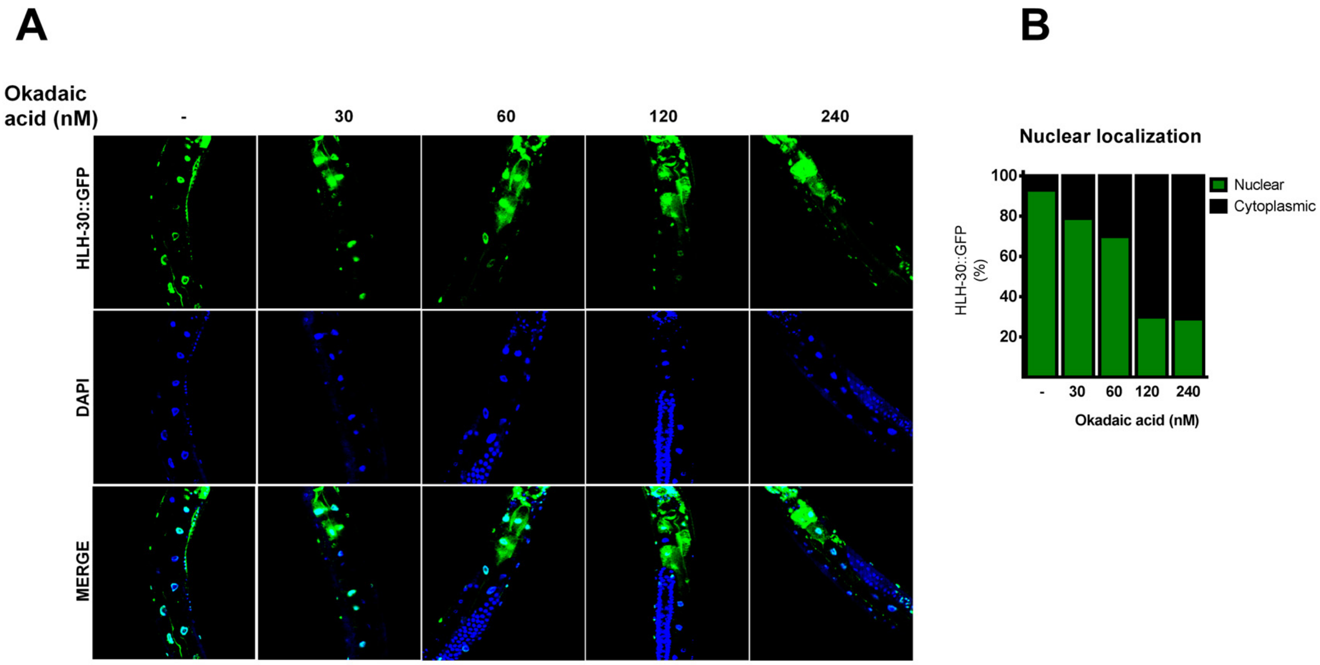

Supplementary Figure 2. Dose response assay of nuclear localization of HLH-30::GFP. Okadaic acid decreased HLH-30 nuclear localization in a concentration-dependent manner. (**A**) Representative fluorescence images showing HLH-30::GFP in worms fed a high glucose diet and different okadaic acid concentrations (30, 60, 120 and 240 nM) for 24 h. Quantification is given in (**B**). Nuclei were labeled with DAPI (blue dots).
